# Analysis of longitudinal semicontinuous data using marginalized two-part model

**DOI:** 10.1186/s12967-018-1674-5

**Published:** 2018-11-06

**Authors:** Miran A. Jaffa, Mulugeta Gebregziabher, Sara M. Garrett, Deirdre K. Luttrell, Kenneth E. Lipson, Louis M. Luttrell, Ayad A. Jaffa

**Affiliations:** 1 Epidemiology and Population Health Department, Faculty of Health Sciences, American University of Beirut, P.O.Box 11-0236, Riad El-Solh, 1107 2020 Beirut, Lebanon; 2 Department of Biochemistry and Molecular Genetics, Faculty of Medicine, American University of Beirut, Beirut, Lebanon; 3 Department of Public Health Sciences, Medical University of South Carolina, Charleston, SC USA; 4 Department of Medicine, Medical University of South Carolina, Charleston, SC USA; 5 Fibrogen Inc, San Francisco, CA USA

**Keywords:** Connective tissue growth factor, Longitudinal data, Marginalized two-part model, One-part model, Semicontinuous data, Two-part model, Type 1 diabetes

## Abstract

**Background:**

Connective tissue growth factor (CTGF), is a secreted matricellular factor that has been linked to increased risk of cardiovascular disease in diabetic subjects. Despite the biological role of CTGF in diabetes, it still remains unclear how CTGF expression is regulated. In this study, we aim to identify the clinical parameters that modulate plasma CTGF levels measured longitudinally in type 1 diabetic patients over a period of 10 years. A number of patients had negligible measured values of plasma CTGF that formed a point mass at zero, whereas others had high positive values of CTGF that were measured on a continuous scale. The observed combination of excessive zero and continuous positively distributed non-zero values in the CTGF outcome is referred to as semicontinuous data.

**Methods:**

We propose a novel application of a marginalized two-part model (mTP) extended to accommodate longitudinal semicontinuous data in which the marginal mean is expressed in terms of the covariates and estimates of their effect on the mean responses are generated. The continuous component is assumed to follow distributions that stem from the generalized gamma family whereas the binary measure is analyzed using logistic model and both have correlated random effects. Other approaches including the one- and two-part with uncorrelated and correlated random effects models were also applied and their estimates were all compared.

**Results:**

Our results using the mTP model identified intensive glucose control treatment and smoking as clinical factors that were associated with decreased and increased odds of observing non-zero CTGF values respectively. In addition, hemoglobin A1c, systolic blood pressure, and high density lipoprotein were all shown to be significant risk factors that contribute to increasing CTGF levels. These findings were consistently observed under the mTP model but varied with the distributions for the other models. Accuracy and precision of the mTP model was further validated using simulation studies.

**Conclusion:**

The mTP model identified new clinical determinants that modulate the levels of CTGF in diabetic subjects. Applicability of this approach can be extended to other biomarkers measured in patient populations that display a combination of negligible zero and non-zero values.

## Background

Diabetes mellitus is a progressive disease of the vasculature, leading to increased risk of both microvascular complications such as diabetic nephropathy (DN) and retinopathy (DR), and cardiovascular disease (CVD), including myocardial infarction and stroke [1, 2]. Emerging evidence points to a mechanistic link between microvascular complications such as DN and DR and increased risk of cardiovascular disease [3–5]. Since early pathologic events are similar within small and large vessels, it is postulated that common risk markers and mechanisms that initiate and promote vascular damage are involved. One such factor that has been identified as a pathogenic risk determinant for the development of microvascular and cardiovascular complications is connective tissue growth factor (CTGF). CTGF is a secreted matricellular potent chemotactic and extracellular matrix-inducing factor that has been implicated in progression of inflammatory and fibroproliferative disorders [6]. Plasma CTGF levels were independently associated with hypertension, increased albumin excretion rate, increased carotid intima-media thickness, hemoglobin A1c (HbA1c) and circulating levels of lipoproteins [7]. Plasma CTGF was also linked to increased risk of cardiovascular events and mortality in patients with atherosclerotic disease and was associated with plaque stabilization following stroke [8, 9]. Moreover, plasma CTGF levels were shown to predict myocardial infraction in type 2 diabetic subjects [10]. Taken together, these studies suggest that CTGF may have substantial value both as a pathogenic risk marker of inflammation-induced tissue injury and as a therapeutic target.

Despite that the divergent biological effects of CTGF on the vasculature was established, it still remains unclear how CTGF expression is regulated. To gain insights into the factors that modulate plasma CTGF levels, circulating levels of CTGF were measured longitudinally in type 1 diabetic patients over a period of 10 years. Our results indicated that a number of patients had negligible measured values of plasma CTGF that formed a point mass at zero, and other patients had high values of CTGF that were measured on a continuous scale. The combination of excessive zero and continuous positively distributed non-zero values in the CTGF outcome observed in our study is referred to as semicontinuous data [11]. The cause behind the semicontinuous data of plasma CTGF may be attributed to factors or clinical covariates that either promote expression and release of CTGF and/or inhibition of CTGF in diabetic subjects. Hence, it is important to identify the clinical factors that associate with the odds of having detected non-zero CTGF values as well as determining the factors that correlate with CTGF levels. This clinical problem motivated our research work in which we present different models for analysis of the semicontinuous CTGF data considered in this manuscript.

Semicontinuous data is given special attention in the literature due to its widespread occurrence under different settings, and the importance of its appropriate analysis in order to obtain accurate estimates and inferences [12]. Given the mixture of zero and non-zero values, it was intuitive to view the semicontinuous outcome as arising from two different stochastic processes. One process, referred to as the binary part, indicates if the outcome is zero or not, and the second referred to as the continuous part, determines the positive values conditional on the outcome being non-zero. Semicontinuous data are typically analyzed using two-part models wherein the zero process and the continuous values are modeled separately using logistic regression for the binary part and log-normal for the continuous part to ensure prediction of positive values [12]. To analyze longitudinal semicontinuous data, two frameworks were proposed, the two-part mixed models with either correlated or non-correlated random effects in both parts [11–15] and the other is based on the two-part marginal models [16], in addition to Smith et al. [17] who proposed a Bayesian inferential approach for a marginalized Two-part model with correlated random effects. Interpretation of the covariate estimates depends on the model’s specification. Estimates of the continuous component of the Two-part mixed models are interpreted as having conditional effect on the population average given that the outcome values are positive and non-zeros. However, parameter estimate of a covariate in the continuous part of two-part marginal models, is interpreted as having subject-specific and population average multiplicative effect on the population marginal mean if the corresponding covariate is not a random effect. If intercept is the only parameters included as a random effect in the specification of the overall mean, then all covariates will have a multiplicative effect on the population mean. Traditional approaches such as zero inflated Poisson and zero inflated negative binomial models are mainly implemented to address data with zero mass discrete count outcome and cross-sectional data. Available approaches for semicontinuous data are known to be computationally intensive and sometimes not feasible to implement [15]. Some of the available models involve complex and intractable integration of high dimensional integration over the stochastic processes in the marginal likelihood function rendering them difficult to implement [12]. In this manuscript we present a novel application of different approaches to analyze semicontinuous data with the aim of assessing the effect of clinical parameters on the processes of the zero and non-zero values of CTGF. The approaches considered in this manuscript included marginalized two-part that we extend to accommodate longitudinal repeated measures data, two-part correlated and uncorrelated random effects, and one-part models. These models are advantageous in terms of feasibility of implementation by using available statistical procedures such as SAS proc NLMIXED used for mTP and TP with correlated random effects, and SAS Proc Glimmix for TP with uncorrelated random effects compared to the other available approaches which are complex and computationally intensive [12, 15] and require EM algorithms or Bayesian approaches to be implemented. Another advantage of the mTP when modelled for cross sectional data is that its estimates were consistent and unbiased [18].

To our knowledge, all studies that centered on CTGF as an outcome applied conventional statistical methods that ignore the zero part and just analyze the non-zero continuous part. Ignoring the zeros will not allow for determining the factors that affect the zero values of CTGF, and information from the zero component will not contribute to the likelihood function, which introduces bias to the estimation process.

The Marginalized two-part model implemented here is an extension of a previous work for cross sectional studies by Voronca et al. [18] that we developed to analyze longitudinal semicontinuous data. This extension of the mTP model to longitudinal data with repeated measures imposes an added level of complexity due to the inclusion of random effects that account for the within-subject correlation. In addition, this extended model incorporates higher dimensional variance–covariance matrix that accounts for the correlations between the random effects of the zero and non-zero processes. In specific, the within-subject correlation is accounted for by including correlated random effects in the binary and continuous parts. Random intercepts in both parts are jointly modeled in a marginalized likelihood function integrated over the random effects. Intercepts are the only random effect included in the overall specification of the mean. The marginal mean is parameterized directly in terms of regression coefficients using both zero and non-zero values and direct interpretation of the covariate effects on the marginal mean can be drawn for the entire population and not conditional on the positive values. The generalized gamma family of distributions known for its flexibility to account for different types of data was incorporated in our longitudinal model. In addition, we considered the special cases of the generalized gamma that include standard gamma, Weibull, and lognormal. Generalized gamma distribution is defined by parameters for the shape and scale that give it flexibility and appropriateness to fit datasets with different skewness and asymmetry.

In the model’s section, we start first by describing the generalized gamma family of distributions, and the two-part models for longitudinal data with uncorrelated and correlated random effects. We then describe the marginalized two-part model with generalized gamma and other distributions that stem from this family, and the one-part model that analyzes the whole data with zeros and non-zeros. These models were applied to a cohort of diabetic patients with CTGF measures as the outcome of interest that motivated this study.

### Study population

Plasma CTGF levels were measured on 693 subjects from the Diabetes Control and Complications Trial (DCCT)-cohort of type 1 diabetes [19]. The patients enrolled in the DCCT study between 1983 and 1989 and half of the subject population was randomly assigned to conventional diabetes treatment and the other half was assigned to intensive diabetes treatment. In 1993, the DCCT study was stopped when intensive treatment was clearly shown to reduce the risks of microvascular complications [20]. The DCCT study was approved by the Institutional Review Boards of all participating DCCT centers and all participants provided written informed consent. Clinical factors such as blood pressure, HbA1c, lipoprotein, duration of diabetes, and demographic factors such as age, gender, and smoking were all collected on these patients and were used as covariates in our analysis to assess their effects on CTGF levels.

### CTGF measurement

**Fig. 1:**
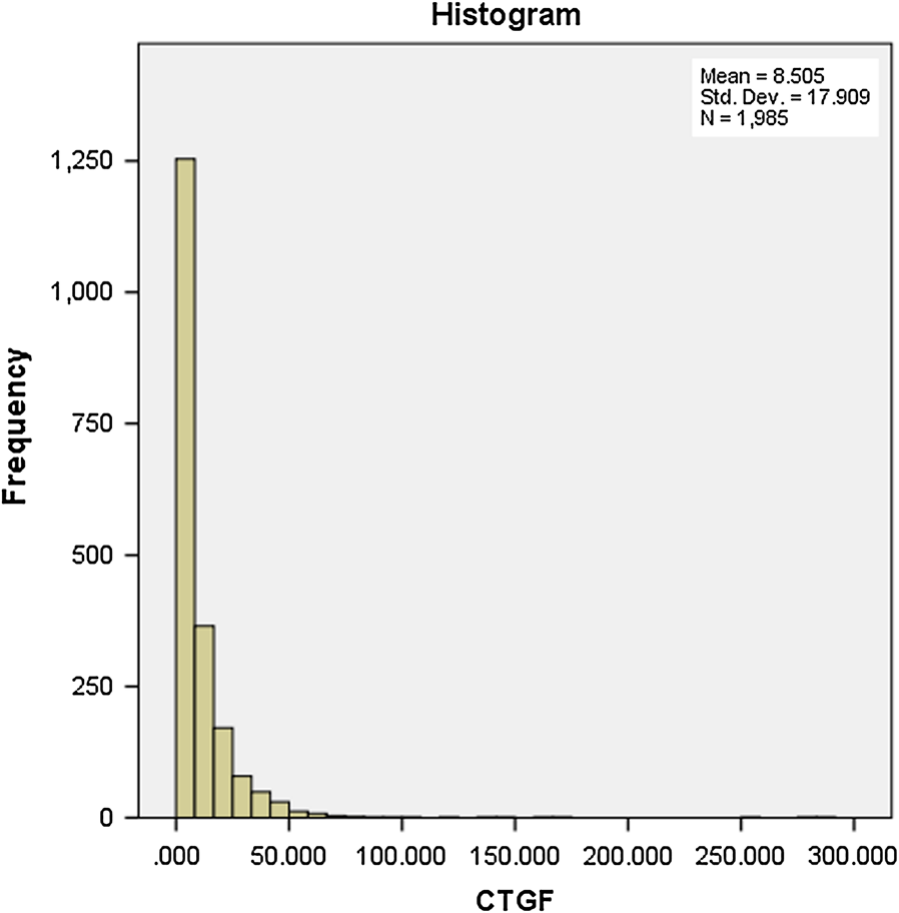
Histogram depicting the frequency of CTGF (ng/ml) levels measured in 693 type 1 diabetic patients

Plasma CTGF levels were measured longitudinally at baseline [study entry (1983–1989)], mid-point of DCCT (1988–1991) and end of DCCT (1993) with a sandwich ELISA that detects both intact CTGF, and cleaved CTGF to release the N-fragment of CTGF (N + W-CTGF assay). The capture antibody is human anti-human CTGF-domain 1 and the detection antibody is mouse anti-human CTGF-domain 2 (FibroGen, San Francisco, CA, USA). Standard curve was prepared with rhCTGF (CTGF expressed in CHO cells and affinity purified with an anti-CTGF antibody column, FibroGen, San Francisco, CA USA). Absorbance at 405 nm was acquired on a SpectraMax 340PC spectrophotometer and analyzed with SoftMax Pro 4.8 software (Molecular Devices, Sunnyvale, CA, USA). The total CTGF values of the repeated measures (n = 1985) in all subjects throughout the study are plotted in Fig. 1. The data shows that about 62% (n = 1231) of CTGF levels measured were negligible and close to zero, suggesting that the production and/or release of CTGF into the plasma is inhibited in subjects with zero measured values of CTGF.

## The models

Four models were explored and illustrated: (1) two-part model for longitudinal data with uncorrelated random effects, (2) two-part model for longitudinal data with correlated random effects, (3) marginalized two-part model, and (4) one-part model. We first start by describing the generalized gamma and the distributions that stem from this family which are the gamma, Weibull and lognormal distributions. These different distributions were considered for the continuous part of the Two-part models and the marginalized two-part model. As for the one-part model, these distributions were applied on the entire sample that has both the zero values and the continuous part altogether.

### Generalized gamma family of distributions

We describe here the modeling framework of the generalized gamma distribution determined by three parameters for the shape and scale. Specifications of these parameters result in certain distributions such as standard gamma, lognormal, and Weibull. Thus, this family of distributions is appropriate to help understand the dependent variable and the process behind generating its values by comparing the model fit for each of the distributions and to select the best estimates using maximum likelihood approach in a regression framework.

The generalized gamma probability density function is specified as such:

1 $$f\left( {y;k,\mu ,\sigma } \right) = \frac{{\eta^{\eta } }}{\sigma y\varGamma \left( \eta \right)\sqrt \eta }\exp \left\{ {u\sqrt \eta - \eta \exp \left( {\left| k \right|u} \right)} \right\},$$

where $\varGamma \left( . \right)$ is the standard gamma function, $u = sign\left( k \right){{\left( {\log y - \mu } \right)} \mathord{\left/ {\vphantom {{\left( {\log y - \mu } \right)} \sigma }} \right. \kern-0pt} \sigma }$, for shape parameter k, location parameter $\mu > 0$ and scale parameter $\sigma > 0$ and $\eta = \left| k \right|^{ - 2} > 0$.

The sth moments of the GG distribution are specified as such:

2 $$E\left( {Y^{s} } \right) = \exp \left\{ {\mu^{s} + \frac{{s\sigma \log \left( {k^{2} } \right)}}{k} + \log \left[ {\varGamma \left( {\frac{1}{{k^{2} }} + \frac{s\sigma }{k}} \right)} \right] - \log \left[ {\varGamma \left( {\frac{1}{{k^{2} }}} \right)} \right]} \right\}$$

And mean and variance are respectively

3 $$E\left( Y \right) = \exp \left\{ {\mu + C\left( {\sigma ,k} \right)} \right\}$$

where $$C\left( {\sigma ,k} \right) = \frac{{\sigma \log \left( {k^{2} } \right)}}{k} + \log \left[ {\varGamma \left( {\frac{1}{{k^{2} }} + \frac{\sigma }{k}} \right)} \right] - \log \left[ {\varGamma \left( {\frac{1}{{k^{2} }}} \right)} \right]$$and

4 $$Var\left( Y \right) = \left\{ {\exp \left( \mu \right)k^{{{\raise0.7ex\hbox{${2\sigma }$} \!\mathord{\left/ {\vphantom {{2\sigma } k}}\right.\kern-0pt} \!\lower0.7ex\hbox{$k$}}}} } \right\}^{2} \left\{ {\frac{{\varGamma \left( {\frac{1}{{k^{2} }} + \frac{2\sigma }{k}} \right)}}{{\varGamma \left( {\frac{1}{{k^{2} }}} \right)}} - \left[ {\frac{{\varGamma \left( {\frac{1}{{k^{2} }} + \frac{2\sigma }{k}} \right)}}{{\varGamma \left( {\frac{1}{{k^{2} }}} \right)}}} \right]^{ - 2} } \right\}$$

Specifications of *σ* and *k* result in different distributions. When *σ* = *k* the gamma distribution is obtained, when *k* = 1 the Weibull distribution is obtained, and when *k* → 0 the limiting distribution of the generalized gamma reduces to lognormal distribution.

### Two-part model for longitudinal data with uncorrelated random effects

The longitudinal two-part model can be described as such:

5 $$g_{TP} \left( {Y_{ij} } \right) = \left\{ \begin{aligned} & 1 - \pi_{ij} \, \quad if \, Y_{ij} = 0 \hfill \\ & \pi_{ij} f\left( {Y_{ij} ;X^{\prime}_{ij} \delta } \right) \, \quad if \, Y_{ij} { > 0 } \hfill \\ \end{aligned} \right.$$

where *Y*_*ij*_ represents the observation for the positive continuous outcome with a point mass at zero for the ith subject at the jth time point, $\pi_{ij} = \Pr \left( {Y_{ij} > 0} \right)$ is the probability of being non zero for the ith subject at the jth time point, and $f\left( {Y_{ij} ;X^{\prime}_{ij} \delta } \right)$ is the density function for the positive values of *Y*_*ij*_. The parameterization of this model is done in two parts that are fit separately:

In part 1 the binary outcome is modeled as

6 $$logit\left( {\Pr \left( {Y_{ij} > 0} \right)} \right) = \log it\left( {\pi_{ij} } \right) = Z^{\prime}_{ij} \alpha + b_{1i}$$

where *b*_1*i*_ represents the random effect intercept that accounts for the within subject correlation pertaining to the repeated measures for the same subject in the zero part

7 $$b_{1i} = N\left( {0,\sigma_{b1}^{2} } \right)$$

Assuming that the log for the *g* link function, the location parameter *μ*_*ij*_ for the continuous component is modeled in the second part as

8 $$g\left( {E\left( {Y_{ij} |Y_{ij} > 0} \right)} \right) = \log \left( {\mu_{ij} |Y_{ij} > 0} \right) = X^{\prime}_{ij} \delta + b_{2i}$$

where *b*_2*i*_ represents the random effect intercept that accounts for the within subject correlation pertaining to the repeated measures for the same subject in the continuous part

9 $$b_{2i} = N\left( {0,\sigma_{b2}^{2} } \right)$$

The two random effect intercepts *b*_1*i*_ and *b*_2*i*_ in the two process of zero and non-zero are assumed to be independent and uncorrelated. $Z^{\prime}_{ij}$ is the vector of covariates for the ith subject measured at the jth time point for the binary part and $X^{\prime}_{ij}$ is the vector of covariates for the ith subject measured at the jth time point used for the continuous part. The two parts might have common covariates or completely different ones. *α* is the vector of model coefficients corresponding to the binary part and *δ* is the vector of coefficients corresponding to the continuous part conditional on the values being non-zero.

The marginal mean and variance of *Y*_*ij*_ from a TP model can be derived as such:

10 $$E\left( {Y_{ij} } \right) = \pi_{ij} E\left( {\left. {Y_{ij} } \right|Y_{ij} > 0} \right), \, Var\left( {Y_{ij} } \right) = \pi_{ij} \left[ {E\left( {\left. {Y_{ij}^{2} } \right|Y_{ij} > 0} \right) - \pi_{ij} E\left( {\left. {Y_{ij} } \right|Y_{ij} > 0} \right)^{2} } \right]$$

When GG is assumed in the continuous part, the marginal mean is

11 $$E\left( {Y_{ij} } \right) = \pi_{ij} \exp \left\{ {\mu_{ij} + C\left( {\sigma ,k} \right)} \right\} = \frac{1}{{1 + \exp \left( { - z^{\prime}_{ij} \alpha } \right)}}\exp \left\{ {X^{\prime}_{ij} \delta + b_{2i} + C\left( {\sigma ,k} \right)} \right\}$$

The variance of *Y*_*ij*_ corresponding to TP can be obtained using the variance formula in Eq. (10) and the sth moments for GG in Eq. (2). C is defined in Eq. (3) and its specification leads to the different distributions that belong to the GG family of distributions. For instance, when *C* = 0 and *σ* = *k* then the GG distribution reduces to the TP-gamma distribution model for the continuous part; when $C\left( \sigma \right) = \log \left[ {\varGamma \left( {1 + \sigma } \right)} \right]$ and *k* = 1 then the TP-Weibull distribution is obtained, and when $C\left( \sigma \right) = {{\sigma^{2} } \mathord{\left/ {\vphantom {{\sigma^{2} } 2}} \right. \kern-0pt} 2}$ and *k* → 0 then the TP-lognormal distribution is obtained.

In the binary part, the estimates of the vector of coefficients *α* represent population based averages for the whole population for the probability of positive values. When taken on an exponential scale, $\exp \left( \alpha \right)$ can be interpreted as the odds ratio of having positive value for a one unit increase in the corresponding covariate. Meanwhile, in the continuous part the vector of coefficients *δ* are estimated for only those with positive non-zero values that represent a portion of the data and not the whole sample. When the log link is assumed in the continuous part, then conditional on the observation being non-zero, the exponential of the estimate of *δ* is the multiplicative change in the value of the outcome when the corresponding covariate increases by one unit. Hence, the binary part provides population estimates for the probability of non-zero, and the continuous part provides estimates for the effect on the population mean given that the value is non-zero.

### Two-part model for longitudinal data with correlated random effects

So far it was assumed that the intercepts in the two processes are the only random effect specified in the two-part model and that these random variables are independent. This assumption of independence leads to biased estimates in the regression coefficients and the variance components in the continuous part [21]. To correct this assumption the two random effects are assumed to be dependent and their correlation is included in the model specification and likelihood function. In this case the random effects are assumed to have joint distribution which could be the bivariate normal distribution determined as such:

$$b_{i} = \left[ \begin{aligned} b_{1i} \hfill \\ b_{2i} \hfill \\ \end{aligned} \right] = BVN\left( {\left[ {\begin{array}{*{20}c} 0 \\ 0 \\ \end{array} } \right],\varSigma = \left[ {\begin{array}{*{20}c} {\sigma_{b1}^{2} } & {\sigma_{b12}^{2} } \\ {\sigma_{b12}^{2} } & {\sigma_{b2}^{2} } \\ \end{array} } \right]} \right)$$

The binary part provides subject-specific estimates for the probability of obtaining non-zero values, and the continuous part provides subject-specific estimates of the conditional mean of log of the outcome provided the value is non-zero.

The estimation of *Z*_*i*_, *X*_*i*_, and *Σ* can be estimated by maximizing the marginal of the log likelihood function that is integrated over the random effects that can be described as such:

12 $$L = \prod\limits_{i} {\int {\left( {\left( {1 - \pi_{ij} |Z_{i} ,b_{1i} } \right)^{{I\left( {Y_{ij} = 0} \right)}} \left( {\pi_{ij} |Z_{i} ,b_{1i} } \right)^{{I\left( {Y_{ij} > 0} \right)}} f\left( {Y_{ij} |X_{i} ,b_{2i} } \right)^{{I\left( {Y_{ij} > 0} \right)}} \theta \left( {b_{i} |\varSigma } \right)d_{{b_{i} }} } \right)} }$$

where *f* represents the distribution function of the continuous part of the outcome *Y*, and *θ* represents the bivariate normal distribution for the random intercepts.

### Marginalized two-part models (MTP) extended to longitudinal data

The longitudinal form of the probability density function (pdf) for an MTP model $\left( {g_{MTP} } \right)$ can be written as such:

13 $$g_{MTP} \left( {Y_{ij} } \right) = \left\{ \begin{aligned} & 1 - \pi_{ij} \, \quad if \, Y_{ij} = 0 \hfill \\ & \pi_{ij} f\left( {Y_{ij} ;X^{\prime}_{ij} \beta ,u_{2i} } \right) \quad\, if \, Y_{ij} >; 0 \, \hfill \\ \end{aligned} \right.$$

where *π*_*ij*_ is the probability of non-zero value for the outcome *Y*_*ij*_ and is obtained from a logistic model, thus it will take the form of $\pi_{ij} = \frac{{\exp \left( {Z^{\prime}_{ij} \alpha + u_{1i} } \right)}}{{1 + \exp \left( {Z^{\prime}_{ij} \alpha + u_{1i} } \right)}}$ and *β* representing the vector of marginal coefficients corresponding to the continuous part of an MTP model, *u*_*i*_ represents the correlated random effect intercepts in both parts of MTP

14 $$u_{i} = \left[ \begin{aligned} u_{1i} \hfill \\ u_{2i} \hfill \\ \end{aligned} \right] \sim BVN\left( {\left[ {0,0} \right],G = \left[ {\begin{array}{*{20}c} {\sigma_{u1}^{2} } & {\sigma_{u12}^{2} } \\ {\sigma_{u12}^{2} } & {\sigma_{u2}^{2} } \\ \end{array} } \right]} \right)$$

The marginal mean is of the form

15 $$E{\text{ (}}Y_{{{\text{ij}}}} {\text{) = exp (}}X^{\prime}_{{\text{i}}} \beta {\text{ + }}u_{{{\text{2i}}}} {\text{) = }}\xi$$

Solving for the location parameter of the GG distribution in *E*(*Y*_*ij*_) expressed in Eq. (11), we get the following parameterization:

16 $$\mu_{ij} = X^{\prime}_{ij} \beta + u_{2i} - \log \left( {\pi_{ij} } \right) - C\left( {\sigma ,k} \right)$$

C is defined in Eq. (3) and its specification leads to the different distributions that belong to the GG family of distributions. For instance, when *C* = 0, and *σ* = *k* the GG distribution reduces to the MTP-gamma distribution model for the continuous part, when $C\left( \sigma \right) = \log \left[ {\varGamma \left( {1 + \sigma } \right)} \right]$, and *k* = 1 the MTP-Weibull distribution is obtained, and when $C\left( \sigma \right) = {{\sigma^{2} } \mathord{\left/ {\vphantom {{\sigma^{2} } 2}} \right. \kern-0pt} 2}$ and *k* → 0 the MTP-lognormal distribution is obtained. The distribution used in the continuous non-zero part of MTP should have a finite closed-form mean that can be parameterized as in Eq. (16).

The binary part provides subject-specific estimates of the probability of having non-zero values for the outcome wherein the exponential of *α* is interpreted as the subject-specific odds ratio for having a non-zero response attributed to a one unit increase in the respective covariate. The continuous part provides effects of the estimates on subject-specific and population mean for parameters corresponding to covariates that are not included as random effects in the model’s specification. This specification was assumed in the correlated mTP model described earlier. Parameter estimates in the continuous part will only have subject-specific interpretation if the corresponding covariates are included as random effects. The exponential of the parameter *β* in the continuous part represents the multiplicative effect on the overall mean for the whole population attributed to a one unit increase in the corresponding covariate *X*. The continuous component of the correlated marginalized Two-part model provides effects of the estimates on the entire sample, while that of the correlated Two-part model provides estimates of the effect on portion of the sample pertaining to the positive non-zero values.

### Statistical estimation and inference for MTP longitudinal models

The general format of the likelihood function can be described as such:

17 $${\text{L}}\left( {\pi ,\mu , {\text{k,}}\sigma , {\text{G|y}}} \right) = \mathop \varPi \limits_{i} \left( {1 - \pi_{ij} |Z_{i} ,u_{1i} } \right)^{{I\left( {Y_{ij} = 0} \right)}} \left\{ {\left( {\pi_{ij} |Z_{i} ,u_{1i} } \right)f\left( {Y_{ij} ;k,\mu_{ij} ,\sigma ,u_{2i} } \right)} \right\}^{{I\left( {Y_{ij} > 0} \right)}} q\left( {u_{i} |G} \right)$$

where *f* represents the pdf of the GG distribution or any other distribution from its family and *q* is the bivariate normal distribution for the random intercepts. Expressing *μ*_*ij*_ in terms of Eq. (16), and *π*_*ij*_ as denoted earlier, the marginal likelihood function for the GG distribution can be described as such

18 $$\begin{aligned} & {\text{L}}\left( {\alpha ,\beta , {\text{k,}}\sigma , {\text{G|y}}} \right) = \mathop \prod \limits_{i} \int {\left( {1 - \frac{1}{{1 + \exp \left( { - Z^{\prime}_{ij} \alpha + u_{1i} } \right)}}} \right)^{{I\left( {Y_{ij} = 0} \right)}} *} \, \hfill \\ & \left\{ {\frac{{\left| k \right|^{{ - 2\left| k \right|^{ - 2} }} }}{{\left( {1 + \exp \left( { - Z^{\prime}_{ij} \alpha + u_{1i} } \right)} \right)\sigma Y_{ij} \varGamma \left( {\left| k \right|^{ - 2} } \right)\sqrt {\left| k \right|^{ - 2} } }}\exp \left\{ {Q\left( {\alpha ,\beta ,\sigma ,k} \right)} \right\}} \right\}^{{I\left( {Y_{ij} > 0} \right)}} q\left( {u_{i} } \right)du_{i} \hfill \\ \end{aligned}$$

where

$$\begin{aligned} Q\left( {\alpha ,\beta ,k,\sigma } \right) = {{sign\left( k \right)\left( {\log Y_{ij} - \left( {X^{\prime}_{ij} \beta + u_{2i} - \log \left( {\exp it\left( {Z^{\prime}_{ij} \alpha + u_{1i} } \right) - C\left( {\sigma ,k} \right)} \right)} \right)} \right)} \mathord{\left/ {\vphantom {{sign\left( k \right)\left( {\log Y_{ij} - \left( {X^{\prime}_{ij} \beta + u_{2i} - \log \left( {\exp it\left( {Z^{\prime}_{ij} \alpha + u_{1i} } \right) - C\left( {\sigma ,k} \right)} \right)} \right)} \right)} {\sigma \sqrt {\left| k \right|^{ - 2} } }}} \right. \kern-0pt} {\sigma \sqrt {\left| k \right|^{ - 2} } }} \hfill \\ - \left| k \right|^{ - 2} \exp \left( {{{k\left( {\log Y_{ij} - \left( {X^{\prime}_{ij} \beta + u_{2i} - \log \left( {\exp it\left( {Z^{\prime}_{ij} \alpha + u_{1i} } \right)} \right) - C\left( {\sigma ,k} \right)} \right)} \right)} \mathord{\left/ {\vphantom {{k\left( {\log Y_{ij} - \left( {X^{\prime}_{ij} \beta + u_{2i} - \log \left( {\exp it\left( {Z^{\prime}_{ij} \alpha + u_{1i} } \right)} \right) - C\left( {\sigma ,k} \right)} \right)} \right)} \sigma }} \right. \kern-0pt} \sigma }} \right) \hfill \\ \end{aligned}$$

In this likelihood function (Eq. 18), *q*(*u*_*i*_) represents the bivariate normal distribution for the random effects with mean vector of zeros and variance–covariance matrix G. The random effects *u*_*i*_ are integrated out to get the marginal likelihood function. The log of the marginal likelihood function is then maximized by taking the first derivative with respect to each parameter and setting the equation to zero to obtain the maximum likelihood estimate for each of the fixed effects, *α*, *β*, k, *σ*, G respectively. Empirical Bayes estimators using the adaptive Gaussian quadrature approach [22] was used to obtain predicted values of the random effects *u*_*i*_. The likelihood function for the standard Gamma, lognormal, and Weibull distributions are obtained in a similar manner by just replacing the distribution of the continuous non-zero part by the corresponding probability density function. The asymptotic standard errors are computed using Fisher information after substituting the maximum likelihood estimates for *α*, *β*, *k*, *σ*, *G* corresponding to the MTP-GG model:

19 $$Var\left( {\hat{\alpha },\hat{\beta },\hat{k},\hat{\sigma },\hat{G}} \right) = diag\left\{ {{\rm I}^{ - 1} \left( {\hat{\alpha },\hat{\beta },\hat{k},\hat{\sigma },\hat{G}} \right)} \right\}$$

The marginal likelihood function is maximized using dual Quasi-Newton optimization [23].

### One-part model for longitudinal data

The one-part model does not distinguish between zero and non-zero values in the sense that it assumes that all values are generated from the same process and the concept of having a zero and non-zeros processes as in the Two-part models does not apply here. Hence, the one-part model analyzes both the zeros and non-zeros as one sample and produces parameter estimates for the whole data (for both the zero and non-zero values altogether).

The one-part model can be described as:

20 $$g\left( {E\left( {Y_{ij} } \right)} \right) = \log \left( {\mu_{ij} } \right) = W^{\prime}_{ij} \gamma + b_{0i}$$

where $b_{0i} \sim N\left( {0,\sigma_{{b_{0i} }}^{2} } \right)$, *γ* is the vector of parameters for the fixed effect covariates *W*_*ij*_. The parameter estimates are generated for the entire sample using approaches such as quasi-likelihood generalized linear models that allow fitting of zero values, or by adding a small constant to the zero values [24]. The one-part model provides estimates of the population-based effects of parameters *γ* on the overall marginal mean, with the exponential of *γ* representing the multiplicative change on *E*(*Y*_*ij*_) corresponding to a one unit increase in the respective covariate when the log link is assumed. It was established that this model results in less efficient, imprecise and biased estimates with inflated type 1 error [24, 25].

## Results

The different models, mTP, TP with correlated random intercepts, TP with uncorrelated random intercepts wherein the zero and continuous parts are fit separately, and the one-part model that fits the zero and non-zero values together in one model and assumes that a single process generates these values, were all applied on the CTGF levels measured longitudinally presenting the outcome of interest. The objective was to identify factors that associate with the zero and non-zero processes that generate the CTGF values in order to gain insight on how these levels are regulated.

Three different distributions were assumed for the continuous part of CTGF; gamma, lognormal and Weibull and their corresponding respective results are shown in Tables 1, 2, 3 wherein we included the slope estimates, its standard errors and P-values. The distribution that fits the data the most was the one that had the least measures of fit, Akaike information criterion (AIC), Bayesian information criterion (BIC) and log Likelihood values. Gamma distribution had the lowest AIC, BIC and log likelihood values indicating that it fits best the data for mTP and TP with correlated random intercepts models (Table 4). Our results discussed in detail below indicated that mTP model gives parameter estimates that are consistent across all distributions while the other models had discrepancy in the hypothesis testing and inferences that were dependent on the distribution of the continuous measures. The models that appeared to have increased inconsistent, inaccurate and biased estimates are the one-part and two-part uncorrelated random intercepts models. The low coverage in the confidence interval and the inflated type 1 error in the one-part model and the attributed bias in the estimates under this model and the uncorrelated two-part especially when zero values are prevailing in the data, explain some of the contradictory results observed in this study.

**Table 1:** Parameter estimates for one-part model, two-part (TP) model with uncorrelated random effects, TP with correlated random effects, and marginalized two-part (mTP) models assuming gamma distribution for the non-zero component

Model component	Covariate	One-part model^a,b^: parameter estimate, (SE), P-value	TP model uncorrelated random effects^c^: Parameter estimate, (SE), P-value	TP model correlated random effects^d^: Parameter estimate, (SE), P-value	mTP model^e^: Parameter estimate, (SE), P-value
Zero part	Intercept	1.4790, (1.0269), 0.1498	0.5006, (0.1246), < 0.0001	− 0.8797, (0.1737), < 0.0001	− 0.1172, (0.0896), 0.1917
	Txt group	0.1582, (0.0942), 0.0932	− 0.2506, (0.1496), 0.0944	− 0.6384, (0.2472), 0.0100	− 0.2899, (0.0609), < 0.0001
	Smoking	− 0.2739, (0.2573), 0.2870	0.5177, (0.1944), 0.0080	0.5693, (0.2767), 0.0400	0.6705, (0.0864), < 0.0001
	Time	0.0630, (0.0176), 0.0003	0.0355, (0.0190), 0.0626	− 0.5005, (0.0725), < 0.0001	− 0.0657, (0.0165), < 0.0001
Continuous Non-zero part	Intercept	–	2.5112, (0.3580), < 0.0001	− 1.8443, (0.5939), 0.0020	− 1.9699, (0.4325), < 0.0001
	HbA1c	0.0597, (0.0507), 0.2387	− 0.0065, (0.0179), 0.7164	0.0970, (0.0244), < 0.0001	0.0755, (0.0214), 0.0005
	Age	− 0.0114, (0.0084), 0.1771	− 0.0121, (0.0041), 0.0045	− 0.0018, (0.0057), 0.7476	− 0.0020, (0.0049), 0.6879
	Duration	− 0.0336, (0.0165), 0.0425	− 0.0082, (0.0065), 0.2098	0.0035, (0.0092), 0.7007	0.0030, (0.0081), 0.7072
	SBP	0.0103, (0.0073), 0.1614	0.0047, (0.0024), 0.0534	0.0267, (0.0043), < 0.0001	0.0243, (0.0029), < 0.0001
	Male	0.0662, (0.1111), 0.5515	0.0501, (0.0637), 0.4330	0.0356, (0.0747), 0.6331	0.0099, (0.0782), 0.8985
	HDL	0.0052, (0.0116), 0.6537	0.0058, (0.0024), 0.0195	0.0172, (0.0034), < 0.0001	0.0124, (0.0031), < 0.0001
	Time	–	0.0201, (0.0102), 0.0530	0.0127, (0.0149), 0.3957	− 0.0225, (0.0164), 0.1710
Random effects	Zero part variance	–	1.0528	0.1894	0.4409
	Non zero part variance	–	0.1620	0.2438	0.3357
	Covariance	–	–	0.2149	0.3847

One-part model^a^ fits the entire sample without distinction between zero and non-zero processes, so only one estimate for the intercept and one for time were generated. In one-part model^b^ the parameter estimates for txt group and smoking represent the effect of these covariates on the CTGF levels themselves and not on the probability of non-zero values, unlike the TP and mTP models. TP model uncorrelated random effects^c^ and TP model correlated random effects^d^ generate estimates for the continuous part using only a portion of the sample pertaining to positive non-zero values. mTP model^e^ provides estimates for the parameters in the continuous part for the entire sample (zero and non-zero values)

**Table 2:** Parameter estimates for one-part model, two-part (TP) model with uncorrelated random effects, TP with correlated random effects, and marginalized two-part (mTP) models assuming lognormal distribution for the non-zero component

Model component	Covariate	One-part model^a,b^: parameter estimate, (SE), P-value	TP model uncorrelated random effects^c^: parameter estimate, (SE), P-value	TP model correlated random effects^d^: parameter estimate, (SE), P-value	mTP model^e^: parameter estimate, (SE), P-value
Zero part	Intercept	2.8436, (0.5773), < 0.0001	0.5006, (0.1246), < 0.0001	− 0.3271, (0.1657), 0.0488	− 0.1414, (0.1120), 0.2072
	Txt group	0.1966, (0.1507), 0.1923	− 0.2506, (0.1496), 0.0944	− 0.4389, (0.2148), 0.0414	− 0.3077, (0.1211), 0.0113
	Smoking	0.3783, (0.1731), 0.0289	0.5177, (0.1944), 0.0080	0.7144, (0.2542), 0.0051	0.6825, (0.1752), < 0.0001
	Time	0.0195, (0.0284), 0.4933	0.0355, (0.0190), 0.0626	− 0.3811, (0.0397), < 0.0001	− 0.0802, (0.0192), < 0.0001
Continuous non-zero part	Intercept	–	2.4621, (0.3478), < 0.0001	− 1.9921, (1.1613), 0.0867	− 1.9869, (0.6854), 0.0039
	HbA1c	− 0.0914, (0.0350), 0.0089	− 0.0058, (0.0174), 0.7370	0.0934, (0.0576), 0.1052	0.0877, (0.0381), 0.0218
	Age	− 0.0427, (0.0154), 0.0055	− 0.0119, (0.0039), 0.0029	0.0011, (0.0131), 0.9339	− 0.0001, (0.0086), 0.9884
	Duration	− 0.0071, (0.0203), 0.7254	− 0.0058, (0.0062), 0.3459	0.0105, (0.0207), 0.6130	0.0012, (0.0141), 0.9307
	SBP	0.0074, (0.0049), 0.1290	0.0042, (0.0024), 0.0801	0.0347, (0.0084), < 0.0001	0.0260, (0.0053), < 0.0001
	Male	− 0.0277, (0.0149), 0.8526	0.0544, (0.0596), 0.3630	0.0011, (0.2038), 0.9957	0.0053, (0.1355), 0.9683
	HDL	0.0080, (0.0052), 0.1207	0.0058, (0.0023), 0.0139	0.0206, (0.0078), 0.0092	0.0142, (0.0053), 0.0078
	Time	–	0.0151, (0.0101), 0.1374	0.0125, (0.0340), 0.7140	− 0.0315, (0.0256), 0.2197
Random effects	Zero part variance	–	1.0528	0.8638	1.4051
	Non zero part variance	–	0.0983	1.2669	1.2052
	Covariance	–	–	1.0543	1.3013

One-part model^a^ fits the entire sample without distinction between zero and non-zero processes, so only one estimate for the intercept and one for time were generated. In one-part model^b^ the parameter estimates for txt group and smoking represent the effect of these covariates on the CTGF levels themselves and not on the probability of non-zero values, unlike the TP and mTP models. TP model uncorrelated random effects^c^ and TP model correlated random effects^d^ generate estimates for the continuous part using only a portion of the sample pertaining to positive non-zero values. mTP model^e^ provides estimates for the parameters in the continuous part for the entire sample (zero and non-zero values)

**Table 3:** Parameter estimates for one-part model, two-part (TP) model with uncorrelated random effects, TP with correlated random effects, and marginalized two-part (mTP) models assuming Weibull distribution for the non-zero component

Model component	Covariate	One-part model^a,b^: parameter estimate, (SE), P-value	TP model uncorrelated random effects^c^: parameter estimate, (SE), P-value	TP model correlated random effects^d^: parameter estimate, (SE), P-value	mTP model^e^: parameter estimate, (SE), P-value
Zero part	Intercept	3.8358, (1.9236), 0.0465	0.5006, (0.1246), < 0.0001	− 0.2263, (0.2050), 0.2700	− 0.1352, (0.1160), 0.2265
	Txt group	0.2915, (0.3266), 0.3725	− 0.2506, (0.1496), 0.0944	− 0.3951, (0.2021), 0.0509	− 0.3018, (0.1205), 0.0125
	Smoking	− 0.7732, (0.3887), 0.0471	0.5177, (0.1944), 0.0080	0.5437, (0.2489), 0.0293	0.4978, (0.1708), 0.0037
	Time	− 0.0384, (0.0537), 0.4747	0.0355, (0.0190), 0.0626	− 0.2700, (0.0387), < 0.0001	− 0.0768, (0.0192), < 0.0001
Continuous non-zero part	Intercept	–	2.4153, (0.3739), < 0.0001	− 1.9956, (1.4045), 0.1558	− 1.9886, (0.7085), 0.0051
	HbA1c	− 0.2964, (0.0905), 0.0011	− 0.0076, (0.0187), 0.6844	0.0988, (0.0672), 0.1418	0.0828, (0.0380), 0.0296
	Age	− 0.0552, (0.0241), 0.0221	− 0.0121, (0.0044), 0.0071	0.0028, (0.0169), 0.8644	− 0.0017, (0.0086), 0.8367
	Duration	− 0.0099, (0.0358), 0.7816	− 0.0094, (0.0069), 0.1749	0.0088, (0.0235), 0.7061	0.0003, (0.0141), 0.9832
	SBP	0.0010, (0.0128), 0.4348	0.0055, (0.0025), 0.0303	0.0322, (0.0091), 0.0005	0.0231, (0.0054), < 0.0001
	Male	− 0.7926, (0.3534), 0.0252	0.0453, (0.0673), 0.5010	0.0006, (0.2411), 0.9980	0.0057, (0.1352), 0.9659
	HDL	0.0209, (0.0131), 0.1091	0.0058, (0.0025), 0.0230	0.0193, (0.0094), 0.0400	0.0130, (0.0053), 0.0150
	Time	–	0.0235, (0.0109), 0.0314	0.0099, (0.0373), 0.7888	− 0.0282, (0.0257), 0.2716
Random effects	Zero part variance	–	1.0528	0.6612	1.3971
	Non zero part variance	–	0.2094	1.6636	1.2199
	Covariance	–	–	1.0488	1.3055

One-part model^a^ fits the entire sample without distinction between zero and non-zero processes, so only one estimate for the intercept and one for time were generated. In one-part model^b^ the parameter estimates for txt group and smoking represent the effect of these covariates on the CTGF levels themselves and not on the probability of non-zero values, unlike the TP and mTP models. TP model uncorrelated random effects^c^ and TP model correlated random effects^d^ generate estimates for the continuous part using only a portion of the sample pertaining to positive non-zero values. mTP model^e^ provides estimates for the parameters in the continuous part for the entire sample (zero and non-zero values)

**Table 4:** Model fit comparison for mTP and TP with correlated random intercepts using gamma, lognormal, and Weibull distributions for the none-zero component

	Model	AIC	BIC	− 2 log likelihood
mTP	Gamma	5827.6	5900.2	5795.6
	Lognormal	6161.1	6238.3	6127.1
	Weibull	6151.3	6223.9	6119.3
TP with correlated intercepts	Gamma	4279.0	4351.6	4247.0
	Lognormal	5186.0	5263.2	5152.0
	Weibull	5357.5	5430.1	5325.5

Our results showed that smoking status was significantly associated with an increase in the probability of non-zero values for CTGF. Specifically smokers had higher odds of 1.7–1.96 of getting non-zero levels of CTGF than nonsmokers with P-values ranging between < 0.0001 and 0.04 depending on the model. This result was consistently demonstrated for mTP, TP with uncorrelated and correlated random effects models, and for all 3 distributions. This result suggests that smoking is associated with increased plasma CTGF levels in type 1 diabetic patients. This lends support to previous findings that CTGF expression levels in pulmonary vessels isolated from smokers was higher than those from nonsmokers [26].

With respect to the impact of intensive glycemic treatment, its effect on the probability of non-zero values of CTGF varied between models and distributions. The mTP model consistently demonstrated its significant effects on the non-zero probability across all 3 distributions. Patients that were on intensive glycemic treatment had 1.34 times lower odds of getting non-zero CTGF values compared to patients on standard treatment (P-values were < 0.0001, 0.0113, 0.0125). Hence, mTP model showed that intensive glycemic treatment is associated with increased probability of having negligible CTGF values. However, the effect of intensive glycemic treatment was not consistently observed in the TP model with correlated random effects in all 3 distributions. In Table 1, when the gamma distribution was used for the continuous measure and in Table 2 with the lognormal distribution, intensive glycemic treatment was significantly associated with decreased odds of having non-zero values for CTGF by about 1.6 times compared to patients on conventional glycemic treatment (P-values = 0.01 and 0.04). However, using the Weibull distribution for the continuous part (Table 3), intensive glycemic treatment had a borderline significant effect with P-value of 0.0509. Given that the gamma distribution was the best fit for this data (AIC = 4279 Table 4), one can deduce that the odds of observing zero CTGF values is exp (0.6384) = 1.89 times higher in intensively treated patients compared to those on the conventional arm. On the other hand, the TP model with uncorrelated random effects failed to capture this significant association between the intensive glycemic treatment group and the probability of non-zero values (P-value = 0.0944). The fact that this model ignores the correlation between the two components and fits the zero part separately from the continuous part treating them as two independent entities might have lowered the power of hypothesis testing and introduced bias in the parameter estimates and inaccuracy in the results.

Unlike mTP and TP, one-part model fits the entire sample and assumes that all values are obtained from a single process instead of two different zero and non-zero processes. Hence, parameter estimates under this model for treatment group and smoking represent the effect of these covariates on CTGF levels and not on the probability of non-zero values. Our results showed that under the one-part model treatment group had no effect on CTGF levels and this was consistently demonstrated for all 3 distributions (Tables 1, 2, 3, one-part model). This result is not in agreement with mTP and clinical findings which supported the hypothesis that intensive glucose control regulates and lowers CTGF levels [7, 27]. As for smoking, its effect on CTGF levels were captured under lognormal (P-value = 0.0289) and Weibull (P-value = 0.0471) distributions but not with Gamma distribution. This detected association showed that smoking contributes to decreased levels of CTGF which is not in line with the results from mTP and TP models as well as clinical findings [26]. This inaccuracy in the estimates could be due to the bias and lack of consistency in the parameter estimates attributed to one-part model.

As for the continuous non-zero part, high density lipoprotein (HDL) was consistently associated with the non-zero values of CTGF, and this was demonstrated for the TP and mTP models and in all 3 distributions (all P-values < 0.05), except for the one-part model that failed to show this association and which could be attributed to the low coverage in the confidence interval of this model. When a significant association was captured, patients with higher plasma HDL levels appeared to have increased levels of CTGF. For example, under the gamma distribution and the mTP model, type 1 diabetic patients who have 1 mg/dl higher HDL had about 1.24% (ng/ml) increased levels of CTGF (Table 1, P-value < 0.0001).

Duration of diabetes and gender consistently demonstrated a non-significant effect on the observed CTGF levels across the TP and mTP models with different distributions. However, the one-part model showed that duration of diabetes, and gender were significantly associated with CTGF only under the gamma and Weibull distributions respectively. These significant associations could be attributed to the fact that the one-part model has increased biased in its estimates and inflated type one error that lead to inaccurate conclusion of significant association when in reality a correlation does not exist. The increased bias and type 1 errors in the one-part model are triggered by the fact that under this approach and unlike the two-part models estimates are generated for the entire sample without distinguishing between the zeros and non-zeros when in fact these values are generated by two different processes.

Systolic blood pressure (SBP) did not have consistently detected effects on the observed values of CTGF in all four models. In this regard, SBP showed a positive significant effect on CTGF under mTP model and TP model with correlated random intercepts for all 3 distributions (P-values < 0.05). A borderline significant effect of SBP on CTGF was detected under TP with uncorrelated random intercepts model with gamma distribution (Table 1, P-value = 0.0534), an insignificant effect with lognormal distribution under these same models (Table 2, P-value = 0.0801), and a significant effect for these models under the Weibull distribution (Table 3, P-value = 0.0303). One-part model showed a non-significant association between SBP and CTGF for all 3 distributions which again could be attributed to the lower coverage in the confidence interval in this model. In addition, given that mTP model and TP model with correlated random effects have less bias in the estimates than one-part and TP with uncorrelated random intercepts models, then one can conclude that SBP has a significant positive effect on the observed non-zero CTGF values. If we were to interpret its marginal effect on the CTGF population mean under mTP model with gamma (Table 1), we can deduce that when SBP increases by 1 mmHg the observed values of CTGF increase by about (exp(0.0243) − 1)*100% = 3% (ng/ml) on average.

When the effect of age on CTGF values was assessed, it also had inconsistent relationship with CTGF observed values that varied with each model. Both mTP and TP models with correlated random effects indicated a nonsignificant association between age and CTGF under all distributions. TP with uncorrelated random effects and one-part models showed significant effect of age on CTGF values (P-value < 0.05) for all distributions under the TP uncorrelated model, and for lognormal and Weibull under the one-part model. These significant results could be attributed to the inflation of type 1 error in the one-part model and TP model with uncorrelated random effects.

Our results also showed that HbA1c, a marker of metabolic control, was significantly associated with CTGF under mTP model with all 3 distributions. In this respect, an increase of 8 ng/ml in the marginal mean of CTGF was attributed to a 1% increase in HbA1c (P-value = 0.0005) under the gamma distribution. Similar interpretation can be drawn for mTP with lognormal and Weibull distributions. The TP model with correlated random effects showed a significant effect of HbA1c only with gamma distribution but did not capture any significant effect with the other distributions. This could be attributed to the fact that mTP had better precision and accuracy in the parameter estimates compared to all other models.

The TP model with uncorrelated random effects did not show any significant association for HbA1c with CTGF. This could be triggered by the decreased power in the hypothesis testing when the zero values are not incorporated in the analysis but rather analyzed separately. One-part model showed a significant negative association between HbA1c and CTGF under the lognormal and Weibull distributions which contradicts the hypothesized positive correlation between HbA1c and CTGF. Lack of precision and increased bias in the one-part model might have led to this inconsistent and inaccurate inference on the direction of the association between HbA1c and CTGF. Hence as previously indicated, our results suggested that mTP exhibited more accurate, precise and consistent estimates and inferences compared to the other models. This conclusion was further examined using a simulation study that intended to determine the performance of each of the models and which we discuss in the following section.

### Simulation study

To assess the performance of each of the models: mTP, TP with correlated random intercepts, TP with uncorrelated random intercepts, and the one-part model, a simulation study was conducted wherein different proportions of zeros were included. We performed 1000 simulations with sample size of 200 and with 9 repeated measures and 30% proportions of zeros, and with 12 repeated measures with 50% proportion of zeros. The performance of each of the models was determined in terms of bias and mean square errors (MSE) and the smaller these performance indicators the more accurate and precise the model’s estimates are. Our simulation results included in Table 5 indicated that mTP had the smallest bias and MSE compared to the remaining models and under the different zero proportions. In this regard, mTP had 35% decrease in bias and 88.4% decrease in MSE compared to TP with correlated random effects in the simulation study that had 30% proportion of zeros, and 39% decrease in bias and 98% decrease in MSE under the simulation study with 50% proportion of zeros. Hence mTP performed better than the TP with correlated random effect and this was evident in both studies especially in the case of higher zero proportion of 50%. In the simulation study with 30% zero proportion, TP with correlated random effect had a decrease of 7% to 12% in bias, and 18% to 21% in MSE compared to TP with uncorrelated random intercepts and the one-part model respectively. Similarly, under the simulation study of 50% zero proportion, a decrease of 2% and 9% was denoted for bias and 8% to 16% in MSE compared to the TP with uncorrelated random intercepts and the one-part model respectively. Hence, our simulation study results suggested that TP with correlated random effects had better performance than the remaining two models (TP with uncorrelated random effects and one-part model), and that mTP had smaller attributed bias and MSE compared to the other 3 models indicating better accuracy and precision of its estimates.

**Table 5:** Simulation results for mTP, TP with correlated random intercepts, TP with uncorrelated random intercepts, and one-part model using simulated data with (a) proportion of zeros is 30% and (b) proportions of zeros is 50%

Model	(a) 30% zero proportion		(b) 50% zero proportion	
	Bias*10	MSE*10	Bias*10	MSE*10
mTP	0.0914	0.0022	0.0915	0.0025
TP with correlated intercepts	− 0.1416	0.1903	− 0.1490	0.2134
TP with uncorrelated intercepts	− 0.1522	0.232	− 0.1522	0.232
One-part	− 0.1671	0.246	− 0.1692	0.253

1000 simulations with sample size of 200 were generated with (a) 9 repeated measures and (b) 12 repeated measures

## Discussion

In this manuscript, we present a novel application of a likelihood-based approach to analyze semicontinuous longitudinal data using a marginalized two-part model that we extended to incorporate longitudinal repeated measures. Various distributions were incorporated that included gamma, lognormal and Weibull. Random intercepts at an individual patient level were introduced in both the zero and non-zero components to account for the within subject correlation inherent due to the repeated measures on the same subject. We applied this model on a cohort of type 1 diabetic subjects with the aim of identifying clinical determinants that associate with CTGF, a pathogenic risk factor for diabetic complications. CTGF levels measured in this cohort displayed a mixture of negligible low values forming a point mass at zero and continuous observed positive values. The objective of this study is to determine what risk factors impact, these two components that ultimately result in CTGF levels. We also compared the estimates under different distributions using other models for analyzing semicontinuous longitudinal outcomes. The models explored here included in addition to marginalized two-part model, the two-part model with correlated, and uncorrelated random effects wherein, the continuous and zero components are fit separately, and the one-part model that provides estimates for the entire sample without distinguishing between the zero and non-zero processes. The marginalized two-part model allows for interpretation of the estimate in the continuous part as 1 unit increase in the covariate on the overall marginal mean comprised of zeros and non-zeros, while the effect of the estimates in the continuous part under the two-part model are interpreted conditional upon the values being observed.

When the mTP model was applied on a cohort of type 1 diabetic patients, it gave consistent results for the parameter estimates across all 3 distributions, demonstrating robustness for the underlying distribution compared to one-part and two-part models with uncorrelated random intercepts. The clinical determinants that displayed significant associations with the probability of non-zero values for CTGF under the mTP model were the glycemic treatment and smoking status. However, the clinical parameters that were significantly associated with the continuous observed positive values of CTGF were HDL, HbA1c and SBP.

In general, the TP model with correlated random effects resulted in estimates that are close to the parameter estimates under the mTP model but showed some discrepancy in the results of some clinical parameters that varied between the different distributions. Specifically, HbA1c was shown to be significantly associated with continuous observed values for CTGF under the TP correlated random intercepts with gamma distribution, but this association was not significant under the lognormal and Weibull distributions. Similarly, the intensive glycemic treatment group was shown to be significantly associated with the probability of non-zero under the gamma and lognormal distributions, but this association was not significant under the Weibull distribution. Gamma and lognormal distributions were better fit for this data given their lower AIB and BIC values and resulted in more stable results than TP with Weibull. This inconsistency in the inferences in the TP model with correlated random intercepts, could be attributed to its sensitivity to the underlying distribution and the true random effects structure, which is not the case with the mTP model [15]. It is worth noting here that from a clinical perspective, HbA1c was shown in some studies to be positively associated with CTGF levels, and treatment was shown to be correlated with the detection levels for CTGF [7, 27].

With respect to the TP model with uncorrelated random intercepts and separate fitting of the zero and continuous components, this approach was consistent with the mTP and the TP models with correlated random intercepts in the inference for smoking that was shown to be significantly associated with the probability of non-zero. However, this was not the case with the parameter estimate for intensive glycemic treatment in the zero part, wherein this model failed to capture the significant effect of intensive treatment on the probability of non-zero measures. Similar result was obtained with HbA1c where a non-significant association was reached under this model with all distributions. SBP was shown to be significantly associated with the continuous part under the Weibull distribution, but not with lognormal or gamma distributions. This result is not in line with findings from other clinical studies that showed a significant association between hypertension and CTGF [7, 9]. The discrepancy in the inferences between the mTP model and the TP model with uncorrelated random effect could be attributed to the increased lack of efficiency, and bias in the parameter estimates due to ignoring the correlation between the random effects in the two components and fitting the zero and continuous parts separately [21, 28].

The one-part model produced estimates and inferences that contradicted clinical findings. For instance this model suggested that increased HbA1c and smoking are protective factors that contribute to decreasing CTGF which is opposite of what has been clinically demonstrated [26, 27]. In this regard, CTGF levels were shown to be significantly associated with HbA1c in type 1 diabetic patients with nephropathy [27]. The expression of CTGF was also shown to be increased in the kidney and vasculature isolated from animal models of diabetes, implicating a role for hyperglycemia in modulating CTGF expression [29, 30]. Furthermore, hyperglycemia was shown to stimulate the expression of CTGF in mesangial cells, podocytes and vascular smooth muscle cells and this process involved activation of transforming growth factor beta, MAPK kinase pathway and protein kinase C [31–34]. In addition, the one-part and two-part with uncorrelated random effects were the only models that detected a significant association between age and CTGF levels. This inaccuracy in the results could be due to the inflated type 1 error and negative bias that pose major disadvantages of the one-part model [35].

A simulation study was conducted whereby two different proportions of zero values were considered (30% and 50%) to assess the performance of each of these models. Our simulation results showed that mTP had a superior performance in the sense that it had the smallest attributed bias and MSE compared to the other three models, which suggests better accuracy and precision of the estimates under mTP. This is in line with the results obtained from the clinical application previously discussed wherein we denoted that the mTP model generated consistent and robust estimates for the assumptions pertaining to the distributions of the continuous part and also accounts for the longitudinal measures and skewness in the data due to the point mass at zero. An advantage of this model resides in the consistency of the estimates and feasibility of its implementation, unlike most of the available approaches for fitting longitudinal semicontinuous data that are computationally intensive and difficult to implement [15]. Other approaches require high dimensional integrations of the stochastic processes in the marginal likelihood function which could be very complex and intractable [12]. As for the execution time, mTP and TP with correlated random effects needed more time to converge, which was about double the time needed for the TP with uncorrelated random effects and the one-part models. This increased execution time is not surprising given the complexity of the likelihood functions and its maximization under mTP and TP with correlated random effects compared to the simpler models of TP uncorrelated random effects that fits the zero and continuous components separately, and the one-part model that fits both components as one sample. However, the overall execution time is still short and it needed less than or approximately 1 min maximum time to converge successfully. Nevertheless, this additional execution time is outweighed by the gain in accuracy and precision of the mTP model.

## Conclusion

In summary, our findings showed that mTP provided stable estimates that are less sensitive to the underlying distributions when compared to the two-part and one-part models. Our simulation results showed superiority of mTP over the other models in terms of minimum bias and mean square errors indicating better accuracy and precision of the parameters’ estimates. Incorporating the within-subject correlation and the correlation between the zero and continuous non-zero processes and expressing the marginal mean directly in terms of parametrization of the regression coefficients using both the zero and non-zero values could all contribute to the precision and accuracy of this model.

Furthermore, in this manuscript we adopted a novel approach that analyzes for the first time CTGF from the perspective of having different processes that result in the zero and non-zero values. The mTP model presented here has identified new clinical determinants that modulate the levels of CTGF in diabetic subjects. In this regard, intensive glycemic treatment was shown to be associated with decreased odds of CTGF detection, and smoking was identified as a factor that associates with increased probability of non-zero which indicates its association with increased levels of CTGF. Moreover, HDL, SBP and HbA1c were associated with increased levels of CTGF. This finding is of clinical significance, since it provides insights into factors that affect the levels of CTGF, a pathogenic risk factor for diabetic complications. In addition, a key advantage is that the analytical approaches described herein are applicable to all inflammatory biomarkers and cytokine profiles measured in patient populations that display a combination of negligible zero and non-zero values to understand the factors that regulate their production. Moreover, the models illustrated in this study are not limited to only clinical outcome datasets but could be also applicable to a vast array of real life situations such as health services research whereby lack or absence of a service may lead to proliferation of zero values, which requires this type of analyses. Hence, this study could be utilized as a model approach for the analyses of similar settings wherein semicontinuous data is present.

## Abbreviations

CTGF: connective tissue growth factor
CVD: cardiovascular disease
mTP: marginalized two-part model
TP: two-part model
AIC: Akaike information criterion
BIC: Bayesian information criterion
SBP: systolic blood pressure
HDL: high density lipoprotein
HbA1c: hemoglobin A1c
